# Local Anesthesia Onset and Pain Perception in Hemophilic and Thalassemic Conditions

**DOI:** 10.3390/jcm12113646

**Published:** 2023-05-24

**Authors:** Supriya Das, Shashirekha Govind, Debkant Jena, Sumit Dash, Siba Prasad Jena, Deepika Yadav, Smita Karan, Jyothsna Kancherla, Amit Jena, Lora Mishra, Sourav Chandra Bidyasagar Bal, Satabdi Pattanaik

**Affiliations:** 1 Department of Conservative Dentistry and Endodontics, Institute of Dental Sciences, Siksha ‘O’ Anusandhan (Deemed to Be) University, Bhubaneswar 751003, Odisha, India; drsupriya93@gmail.com (S.D.); debkantjena@soa.ac.in (D.J.); sumitdash@soa.ac.in (S.D.); sibaprasadjena@soa.ac.in (S.P.J.); yadavdrdeepika@gmail.com (D.Y.); loramishra@soa.ac.in (L.M.); satabdipattanaik@soa.ac.in (S.P.); 2 Department of Dentistry, Shadan Institute of Medical Sciences Research Centre and Teaching Hospital, Himayat Sagar, Hyderabad 500086, Telangana, India; drsmita1975@gmail.com; 3 Department of Dentistry, Dr.V.R.K. Women’s Medical College Teaching Hospital and Research Centre, Aziz nagar, Hyderabad 500075, Telangana, India; jyothu26@gmail.com; 4 Department of Conservative Dentistry and Endodontics, Sriram Chandra Bhanja Dental College & Hospital, Cuttack 753007, Odisha, India; 5 Department of Public Health Dentistry, Institute of Dental Sciences, Siksha ‘O’ Anusandhan (Deemed to Be) University, Bhubaneswar 751003, Odisha, India; souravchandrabidyasagarbal@soa.ac.in

**Keywords:** root canal therapy, irreversible pulpitis, thalassemia, hemophilia, local anesthesia, canal instrumentation

## Abstract

The study aims to evaluate and compare the onset of local anesthesia (LA) and pain perception during endodontic treatment in hemophilic and thalassemic patients. **Methods:** The study included 90 patients with symptomatic irreversible pulpitis of the mandibular molars. Three groups (*n* = 30 in each group) were included. Group 1: hemophilic patients; group 2: thalassemic patients; and group 3: individuals without any systemic diseases. Onset of LA and visual analogue scale (VAS) scores was recorded immediately after the administration of local anesthesia, during the pulp exposure procedure, and during canal instrumentation, and were compared between the three groups. Frequency distribution, ANOVA, and linear regression analysis (*p* < 0.05) were applied. **Results:** The mean onset time was 46 ± 34 s in the hemophilic group, 42 ± 23 s in the thalassemic group, and 38 ± 12 s in controls, but the differences were statistically insignificant. After LA administration (LA-VAS), all three groups experienced a statistically significant reduction in pain (*p* = 0.048). On pulp exposure (PE-VAS) (*p* = 0.82) and during canal instrumentation (CI-VAS) (*p* = 0.55), there was no statistically significant difference in pain perception between the groups. The coefficients indicate a positive correlation between the VAS and onset time, indicating a positive reduction in the VAS following the administration of LA. **Conclusions:** Hemophilic patients exhibited a clinically longer average onset time for LA. However, the difference among the three groups with regard to the overall pain perception after LA administration, during and after pulp exposure, and during canal instrumentation was statistically insignificant.

## 1. Introduction

Endodontists encounter several types of bleeding diseases in routine clinical practice. The earlier identification of the disorders and their potential systemic causes play a major part in minimizing the risk of bleeding during treatment [1]. Hemophilia is a group of inherited disorders characterized by a lack of one or more clotting factors, leading to a prolonged clotting time and potentially catastrophic bleeding tendencies [1]. Thalassemia is an inherited chronic microcytic anemia characterized by defective hemoglobin synthesis and ineffective erythropoiesis [2,3]; it is one of the most confusing hemoglobinopathies and the severity varies from minor anemia to transfusion dependence [4]. The aforementioned conditions challenge the skills of clinicians in view of the fact that severe bleeding is induced during dental treatments, which might be life threatening at times [5,6]. Patients with thalassemia and hemophilia have impaired oral hygiene as a consequence of their decreased immunity [7]. As a result, these patients tend to acquire caries and periodontal disorders [8].

Non-steroidal anti-inflammatory drugs (NSAIDs) are widely used in the management of pain [9]. However, non-selective inhibition of cyclooxygenase (COX) 1 and 2 increases the risk of bleeding from the upper gastrointestinal tract, decreases platelet aggregation by suppressing COX-1-dependent thromboxane A2, and inhibits the production of gastroprotective prostaglandins primarily formed by COX-1, limiting the use of NSAIDs for pain management in hemophilia and thalassemia patients [10,11]. Paracetamol is considered a safer alternative to NSAIDs in patients with bleeding disorders [11].

Profound pulpal anesthesia (inferior alveolar nerve block is given to achieve pulpal anesthesia for mandibular teeth) is mandatory for the success of root canal treatment [12,13]. Effective pain management facilitates a significant reduction in the anxiety and fear associated with the root canal therapy [14]. Local anesthesia is an integral part of endodontic treatment protocols pertaining to the management of pain in clinical practice [15]. As lignocaine diffuses freely through the interstitial tissues and lipid-rich nerves, it provides a rapid onset [16]. Epinephrine prolongs the duration as well as the depth of anesthesia [17]. In healthy persons, the time taken for the onset of local anesthesia is approximately two to three minutes [18]. However, the onset of local anesthesia or pain perception during endodontic treatments in hemophilic and thalassemic patients has not been discussed in the literature.

Hence, the objective of this study is to evaluate the onset of local anesthesia and pain perception during endodontic procedures in hemophilic and thalassemic patients, and to compare them with healthy individuals. The null hypothesis was that there would be no difference in the onset of local anesthesia and that the pain threshold levels would be greater or similar in hemophilic patients and thalassemic patients compared to healthy individuals.

## 2. Materials and Methods

**Figure 1:**
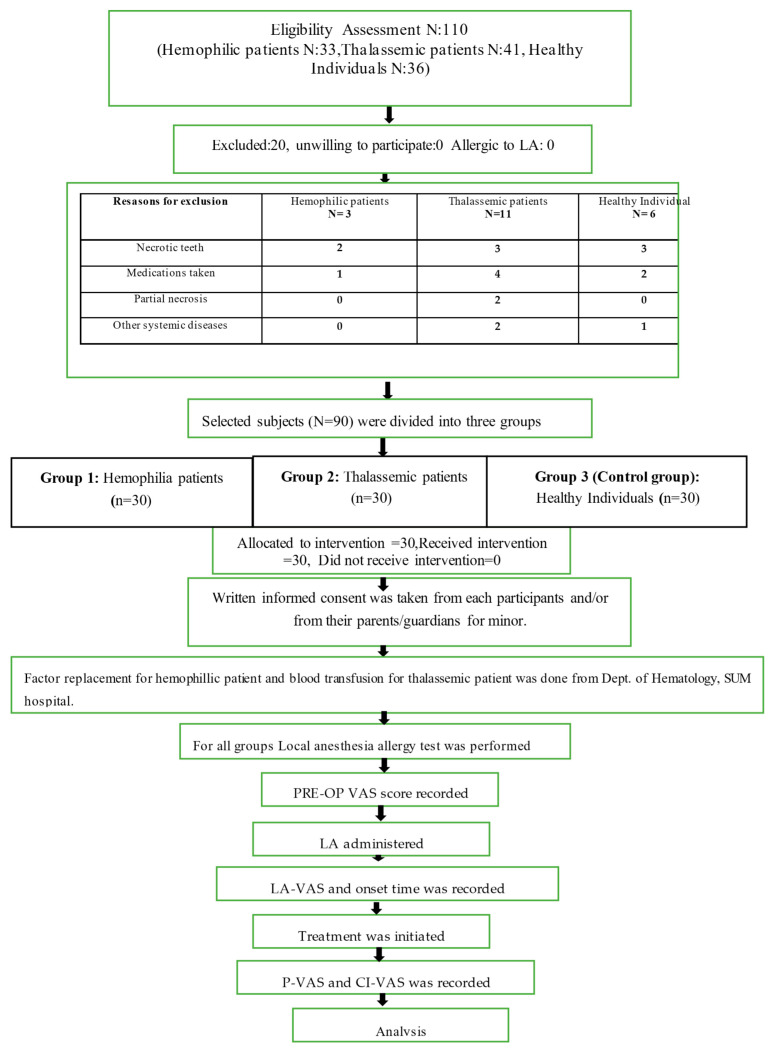
Sample selection and methodology flowchart.

**Figure 2:**
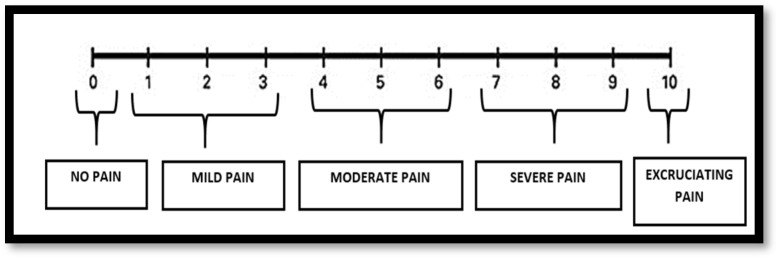
Five-point visual analog scale (NRS).

This study was intended and realized according to the STROBE Statements applicable (URL http//www.strobestatement.org 2018 (accessed on March 2019). The study was approved by the Institutional Ethics Committee of the Institute of Medical Sciences and SUM Hospital, Siksha O Anusandhan (Deemed to be) University, Bhubaneswar, Odisha, India (Ref No/DMR/IMS.SH/SOA/180317), and was conducted in the Department of Conservative Dentistry and Endodontics, Institute of Dental Sciences. Written informed consent was obtained from each participant. The study was conducted during the period 2017–2020. The sample size was determined based on the results from previous studies with [13,19] using G* power software, version 3.1.9 (available at http://www.gpower.hhu.de/en.html (accessed on May 2017)). With the level of significance and the power of the test set at 5% and 80%, respectively, the individual group sample size was *n* = 30 [total 90, standard deviation (SD) = 0.80]. Sample selection and methodology is described in Figure 1. The inclusion criteria were as follows: thalassemic or hemophilic patients above the age of 14 years with a clinical diagnosis of symptomatic irreversible pulpitis, under moderate or severe pain that was documented using a five-point visual analog scale (VAS), (It is a numerical rating scale (NRS), 10-cm in length, marked from 0–10 at an equal spacing of one centimeter. Pain intensity has shown to be highly associated with a 5-point verbal descriptive scale “no pain”, “mild pain”, “moderate pain”, “severe pain”, and “excruciating/worst pain” [20], as shown in Figure 2), and exhibiting a positive response to the electric pulp test (DYE1204128, DENJOY DENTAL Co., Ltd., Changsha, China) and/or cold test (Endo ice, Miracold Plus, Heger Werken, Duisburg, Germany). The study excluded non-vital teeth, teeth with periapical lesions, mentally challenged patients, patients diagnosed with other systemic diseases (e.g., diabetes mellitus, cardiovascular diseases, renal disease, cancer) patients allergic to local anesthesia or sulfites, patients who were taking medications (e.g., anti-depressant, anti-coagulants, antibiotics) that would alter pain perception, pregnancy, and smokers [21].

The methodology is described in Figure 1. This study recorded the detailed case history of the participants. The hemophilic patients underwent factor replacement (Table 1) and the thalassemic patients underwent blood transfusion (Table 2) before endodontic treatment at the Department of Hematology, SUM Hospital, Bhubaneswar, India. On the basis of the inclusion and exclusion criteria, the patients were categorized into three groups (*n* = 30 in each group). Group 1: hemophilic patients; group 2: thalassemic patients; and group 3 (control group): individuals without any systemic diseases. “Preoperative pain” response (Pre-op VAS) was recorded using a visual analog scale (VAS). The local anesthesia allergy test (Septodont, Saint-Maur-des-Fossés, France) was performed for individuals who had no history of LA administration.

Intradermal test [22]: Test solutions were prepared for the patch test by diluting the concentrations of local anesthesia to 1:10 and 1:100 dilutions in the following manner: (a) 1:10 dilution was prepared by drawing 0.1 mL of the full-strength anesthetic solution (2% lignocaine with 1:80,000 adrenalin) into a syringe, placing it into a sterile vial, and then diluting with 0.9 mL of sterile saline; (b) 1:100 dilution was prepared by drawing 0.1 mL of the 1:10 dilution into a syringe, placing it into a sterile vial, and then diluting with 0.9 mL of sterile saline. The injection site (forearms) was cleaned with a sterile alcohol swab and dried. The site was then marked at 3 cm apart. The intradermal injection was performed by inserting a 30-gauge needle tip, bevel up, just underneath the surface of the skin and injecting 0.1 mL of the 1:100 dilution of the agent, forming a “bleb”, and the 1:10 dilution was administered next unless precluded by a significantly positive reaction to the 1:100 dilution. One milliliter of intradermal full-strength anesthetic was administered unless precluded by a significantly positive reaction to the 1:10 dilution. Each injection site was evaluated for 15–20 min for any allergic reactions. If a reaction occurred, a tourniquet was tied above the injection site, appropriate treatment was rendered [22], and the patient was excluded from the study. Non-allergic patients were included in the study.

All individuals underwent standard electric pulp and cold testing to assess tooth vitality. To verify the readings, contralateral tooth responses were recorded and then performed on the suspected tooth. The teeth were cleaned prior to testing and as an interface medium, a tooth paste (Sensodyne, Global Health Care Products, Silvasssa, India) was applied to the buccal crown surface after drying and isolating teeth with a cotton roll. An electric pulp tester (EPT) probe placed on the sound coronal third of the labial surface recorded the patient’s “tingling” sensation. Cold testing with Endo ice (Miracold Plus, Heger Werken, Duisburg, Germany) involved placing a large cotton pellet on the buccal surface of the tooth for 15 s or until the patient responded [23]. The patient response to both tests was recorded. This procedure was performed both before and after local anesthesia. The chronbach’s alpha for EPT was found to be 0.84, indicating a strong reliability of the instrument used.

**Figure 3:**
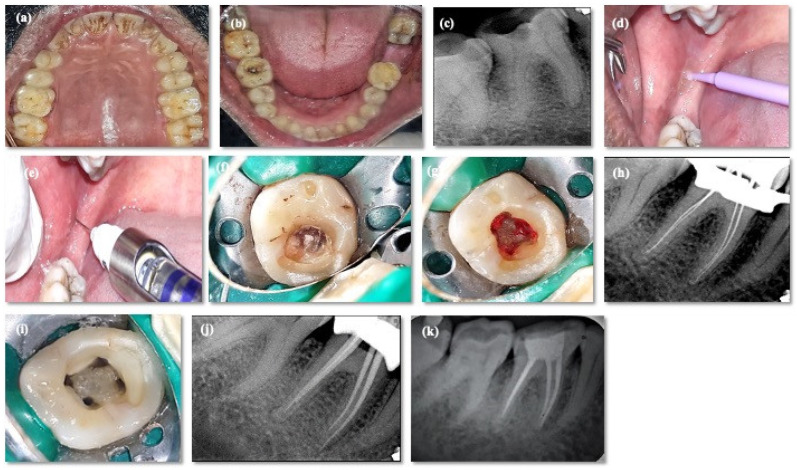
Demonstrating clinical steps representing the groups (hemophilic case). (**a**,**b**) Pre-operative maxillary arch and mandibular arch; (**c**,**f**) pre-operative radiograph and clinical picture i.r.t 46; (**d**) topical anesthesia application; (**e**) inferior alveolar nerve block; (**g**) pulp exposure; (**h**) working length determination; (**i**) after cleaning and shaping; (**j**) master cone selection; and (**k**) post obturation radiograph.

A small quantity of benzocaine gel (20% ProGel-B, Septodont, Saint-Maur-des-Fossés, France) was applied at the injection site using a microbrush applicator for 10–15 s. The aforementioned gel was left in contact with the mucosa for two minutes for effective action. Standard inferior alveolar nerve block (IANB) was administered using a solution comprised of 2% lidocaine with a 1:80,000 epinephrine concentration (Septodont, Saint-Maur-des-Fossés, France) using 1.8 mL Septodont cartridges (Septodont Fusion syringe, Septodont, Cambridge, ON, Canada) and 27-gauge needles (25 mm in length) (Septoject, Septodont, Navi Mumbai, India) over a 1-min time period. The time of onset (in seconds) of action of LA [VAS score (LA-VAS)] was recorded based on initiation of subjective symptoms (lip/tongue numb) by an independent trained evaluator who was blinded to the study protocol. Subsequently, rubber dam isolation of the tooth was performed, and the treatment was initiated within 15 min of LA administration [24]. Successively, access opening was performed using an Endo-Z bur (Dentsply, Gurugram, India), and the VAS score during pulp exposure (PE-VAS) was recorded. A complete de-roofing of the pulp chamber was performed using an Endo-Z bur (Dentsply, India). If pain persisted (VAS ≥ 4) during exposing the pulp or deroofing, the volume of the local anesthetic solution (1.8 mL) was increased using the second cartridge. The VAS score (CI-VAS) was recorded during orifice enlargement and canal instrumentation (CI). If pain continued to persist, a supplementary injection comprised of 2% lidocaine (Septocaine, Septodont, Cambridge, ON, Canada) was administered as a pulpal infiltration if needed. In the absence of pain, the procedure was continued, and the treatment was completed. Root canal procedures were performed by a single operator and were completed in 60 min. Figure 3 depicts clinical procedure of group 1.

Statistical analysis: The data were scrutinized, coded, and analyzed using IBM SPSS statistics 24.0 (SPSS South Asia Pvt Ltd., www.spss.co.in (accessed on April 2023). The descriptive statistics, including the mean and standard deviation of the scale variables, such as age, gender, and volume, were calculated using the frequency distribution procedure. A comparison of the 3 categories, LA-VAS, PE-VAS, and CI-VAS, with PRE-OP VAS and the mean time of onset of the three groups was performed using the analysis of variance (ANOVA) and linear regression analysis. *p* < 0.05 was considered to be statistically significant.

## 3. Results

The hemophilic group had a significantly higher proportion of patients in the age groups of 14–20 years (46.7%) and 21–29 years (36.7%), and the thalassemic group in the age group 30–39 years (36.7) (Table 3). The hemophilic group displayed extremely high male predominance (93.3%) (Table 4).

The comparison of the mean onset time between the groups is shown in Table 5, which implies that the hemophilic group had the longest mean onset time of 45.96 ± 34.32 s and control group had the shortest at 38.96 ± 12.85 s. However, there was no statistically significant difference in the mean onset time between the three groups of patients (*p* = 0.560).

During the pulp exposure procedure, 14 individuals experienced moderate pain, and a repeat volume (1.8 mL) of local anesthetic was administered. As a result, moderate pain was substantially reduced (Table 6).

The comparison of VAS at various intervals is presented in Table 7. The VAS scores between the groups at different interval were statistically insignificant. Pain perception of the three groups at Pre-VAS, LA-VAS, PE-VAS, and CI-VAS is summarized in Table 8. In the preoperative phase (Pre-VAS), the hemophilic, thalassemic, and control groups showed severe pain; however, LA administration (LA-VAS) resulted in a statistically significant reduction (*p* = 0.048) in all three groups. On pulp exposure (PE-VAS) (*p* = 0.82) and during canal instrumentation (CI-VAS) (*p* = 0.55), there was no statistically significant difference in pain perception between the groups.

The regression table (Table 9) indicates that the model is a good fit for predictor values. In LA-VAS, β suggests that for every unit change in onset time, the VAS increases by 1.37 times. In the Pre-op VAS, β is 7.3 indicating a higher VAS due to no treatment. The coefficients indicate a positive correlation between the VAS and onset time, indicating a positive reduction in VAS after LA administration.

## 4. Discussion

Bleeding disorders have always been associated with stigma in the field of dentistry. Hemophilia is the most common inherited bleeding disorder [25]. Conversely, thalassemia is a genetic disorder that involves abnormal hemoglobin formation [26,27]. Some mild forms of thalassemia may cause mild anemia and iron deficiency problems, which might even go unnoticed, while severe forms of thalassemia may even lead to death [2].

Studies have shown that thalassemic patients are susceptible to dental caries because their caries index is high and they have significantly low salivary phosphorous and IgA concentration [4,28]. This occurs because patients are hesitant to visit a dentist for fear of the procedure. According to studies, thalassemic patients are more concerned with their serious medical complications, neglecting their oral health. When decay is too advanced for restorative dental treatments/fillings, thalassemic patients seek dental emergency treatment [8]. Patients with hemophilia also exhibit the same symptoms. According to studies, hemophilic patients are more resistant to dental visits and fear dentists [29]. Patients fear that dental procedures will cause bleeding episodes that can only be controlled by factor infusions, resulting in both physical and financial challenges [29,30]. For the treatment of symptomatic irreversible pulpitis, there is typically no contraindication for endodontic treatment in hemophiliac patients/thalassemic patients. In fact, endodontic treatment is generally preferred over extraction whenever possible, as it does not pose a significant risk of bleeding [1].

In the present study, ≥14 years was included because adolescents currently anticipate a normal average life span and an excellent health-related standard of living [31], and from a dental perspective, complete root formation will be presumed [32]. Regarding treatment, patient comprehension, cooperation, and reciprocity were commendable, and it was observed that the hemophilic group had a significantly higher proportion of patients aged 14–20 and 21–29 years. However, it was found the average age of hemophilic patients was younger than that of thalassemic and control patients. In the studies, elderly individuals develop medical and surgical conditions (such as cancer, prostatic hypertrophy, cardiovascular disease, renal disease, pulmonary hypertension, endocrinological complication) that were not previously observed in younger individuals [33,34].

Pain measurement in these two bleeding disorders is challenging as it includes many associated systemic disorders and psychological factors. For pain measurements, the VAS/NRS are proven for quantification [35]. A study in thalassemic patients found that age increased pain regardless of the diagnosis, transfusion status, gender, bone density, chelator type, or iron overload [36], and hemophilic adolescent patients have preserved joints, acute and transient pain, and easily recognize acute bleeds that are treated earlier and resolved compared to adults [37].

In the current study, symptomatic irreversible pulpitis was considered, characterized by a sharp pain upon thermal stimulus, lingering pain (often 30 s or longer after stimulus removal), spontaneity (unprovoked pain), and referred pain. Postural changes, such as lying down or bending over, can worsen pain, and over-the-counter analgesics rarely work. Deep caries, extensive restorations, and pulpal tissue fractures are common causes. Because the inflammation has not reached the periapical tissues, symptomatic irreversible pulpitis may be difficult to diagnose. In such cases, dental history and thermal testing determine pulpal status [38]. The standard treatment for irreversible pulpitis in permanent mature teeth is non-surgical root canal treatment (NSRCT) [39].

In the present study, the oral hygiene of all groups was considerably good and minor additional clinical findings were treated in subsequent appointments. According to a study, the average onset of LA (Lidocaine) is 1–3 min for subjective symptoms and 1–4 min for objective symptoms [18]. Research shows that most patients experience pulpal anesthetic within 10–15 min of receiving conventional IANB [24,40]. To verify the effectiveness of anesthesia, the cold and EPT test was performed, followed by rubber dam isolation. This procedure was completed in 15 min, so access was opened following 15 min of LA administration. The LA allergic test was performed on three patients (one hemophilic and two in the control group) who had no history of LA administration and they all tested negative.

The local anesthetic solution comprising 2% lidocaine with 1:80,000 epinephrine is routinely used in endodontics. The use of a vasoconstrictor improves local hemostasis [41]. There are no constraints regarding the type of local anesthetic agent that can be used in hemophilic or thalassemic patients [1]. Hence, in this study, an IANB was performed using 2% lidocaine with 1:80,000 epinephrine.

Every year, about 15 million people worldwide are affected with thalassemia. In India, above 12,000 babies are born with hemoglobinopathies every year. On average, one in every twenty-five Indians is a thalassemia carrier [4]. Advances in the field of therapy for managing thalassemia major have emerged during the last two decades, resulting in a situation where patients can expect a normal life expectancy [4]. The proportion of patients in the thalassemic group who were older was considerably greater (i.e., above the age of 40 years). Four individuals in both the thalassemic and control groups experienced moderate pain during pulpal exposure. The pain was alleviated after administration of an extra 1.8 mL volume of the local anesthetic solution. This study included moderate to severe preoperative pain patients, and preoperative pain scores did not differ between the three groups.

Hemophilia is inherited X-linked recessive manner and most prevalent bleeding disorder caused by the deficiency of two coagulation factors: factor VIII (FVIII) or factor IX (FIX) [42,43]. In India, the prevalence of hemophilia A and B is 1 in 5000 and 1 in 30,000, respectively [44]. Hemophiliacs have a short life expectancy, according to past research. However, because of the development of safe and successful treatment procedures, hemophilic patients can now have a normal life expectancy [29]. Since hemophilia is a hemorrhagic ailment that usually exclusively affects male patients, this study found a substantial male predominance in the hemophilic group (X-related recessive disease) [25]. Hemophilia A is more common than hemophilia B, and accounts for 80–85 percent of all occurrences of hemophilia in India [1]. All the patients in the hemophilic group were found to have hemophilia A in the current study.

The hemophilic and thalassemic patients in this study experienced clinically longer onset of local anesthetic action than the control group, but difference was statistically insignificant. Using laser doppler flowmetry, researchers measured the pulpal blood flow in healthy individuals and found that 2% lidocaine (1:100,000 epinephrine) reduced (73%) profound pulpal blood flow at 5 min, with a gradual rebound occurring within 65 min [45]. The delay in onset of LA action may be attributable to compromised clotting proteins and the quantity and quality of hemoglobin in hemophilic and thalassemic patients, respectively, resulting in delay in vasoconstriction and blocking sodium ions flux, which inhibits nerve conduction [17].

In clinical studies involving endodontic procedures on patients with irreversible pulpitis, 19 to 56% of patients experienced mild or no discomfort during endodontic access cavity preparation or initial instrumentation on IANB [13]. According to a study, 39 percent of patients with irreversible pulpitis who received conventional IANB with 2% lidocaine remained sensitive to the cold test [46]. In cases of irreversible pulpitis, it may be difficult to achieve effective anesthesia utilizing the inferior alveolar nerve block alone, as suggested by the previous research [13,24]. Pain intensity is a crucial aspect of chronic pain and a key treatment goal, according to surveys of patients [47]. According to the present study, there was no statistically significant difference in VAS scores between the groups at various intervals, but all groups experienced pain relief after LA administration compared to preoperative levels. It was observed that 14 individuals (8 in group 1, 4 in groups 2 and 3) exhibited moderate pain during pulpal exposure; consequently, a volume of 1.8 mL LA was administered as the IANB as a first option [48]. Subsequently, those individuals were free of pain. Therefore, alternative procedures (such as buccal infiltration, interligamentary, intraosseous, and intrapulpal injections) were not required in the event of IANB failure.

In patients with bleeding disorders, avoiding instrumentation beyond the periapex is essential. As a result, extreme caution was used in the current investigation to avoid instrumentation and filling beyond the apical region of the tooth. The decreased anesthetic effects can be attributed to many reasons, including the fact that the IANB does not always result in significant pulpal anesthesia [13,16], as inflamed tissue nerves have altered resting potentials and decreased excitability thresholds [49,50], and irreversible pulpitis has enhanced sodium channel expression. Because of the lower excitability thresholds, local anesthetic agents do not impede impulse transmission, and the tetrodotoxin-resistant (TTXr) class of sodium channels is resistant to the action of local anesthetic agents [50], [51]. The current study is one of the few studies to investigate at the onset of local anesthesia and pain perception during endodontic procedures in thalassemic and hemophilic patients.

Patient–dentist variables constitute the limitations of the current study. Since pain is a subjective variable, its depiction on a VAS is affected by an individual’s emotional state and prior anesthetic administration experience [52]. The speed of the anesthetic procedure is regulated based on the dentist’s knowledge and skill. Ideally, the anesthetic should be administered slowly [17]. It has been demonstrated that nociceptors’ sensitivity is affected not only by the chemical agent employed, but also by the mechanical effects of the injection site, as well as the injection speed and volume [53]. In spite of these limitations, we were able to avoid the potential sources of bias identified by Hogan et al., 2011 [54] by generating and analyzing sufficient intervention sequences. Few clinical trials have been conducted on these individuals, and contradictory clinical results have been observed. Therefore, more research is required to determine the optimal LA solution type and concentration before it can be used consistently.

## 5. Conclusions

In conclusion, hemophilic patients required a clinically longer onset of LA action, followed by thalassemic patients, but this was statistically insignificant. However, the coefficients indicate a positive correlation between the VAS and onset time, indicating a positive reduction in VAS after the administration of LA. In terms of overall pain perception during and after pulp exposure and during canal instrumentation, there was no statistically significant difference. As a rule, prior to endodontic treatment, patients with hemophilia and thalassemia must undergo factor replacement therapy and a blood transfusion, followed by mandatory antibiotic prophylaxis, to prevent complications.

## Figures and Tables

**Table 1:** Details of factor replacement in hemophilic patients.

Case No.	Age/Sex	Tooth No	Hemophilia	Factor Deficient	Blood Group	Severity of Hemophilia	Factor Replacement Per Appointment [IU/dL] [Eloctate]	Frequency
1	20/M	46	A	VIII	A+	Severe	1000 units	Once in every 2 days
2	17/M	36	A	VIII	B+	mild	250 units	3 days/week
3	15/M	36	A	VIII	AB+	mild	250 units	2 days/week
4	19/F	46	A	VIII	AB+	moderate	1000 units	2 days/week
5	25/M	37	A	VIII	AB+	moderate	1000 units	3 days/week
6	30/M	37	A	VIII	A+	Severe	1500 units	3 days/week
7	22/M	47	A	VIII	B+	Severe	1500 units	Once in every 2 days
8	14/M	46	A	VIII	A+	Severe	500 units	Once in every 2 days
9	16/F	36	A	VIII	AB+	moderate	500 units	2 days/week
10	28/M	37	A	VIII	B+	moderate	1000 units	3 days/week
11	27/M	37	A	VIII	B+	mild	500 units	2 days/week
12	30/M	47	A	VIII	AB+	mild	500 units	2 days/week
13	24/M	46	A	VIII	AB+	mild	500 units	2 days/week
14	15/M	37	A	VIII	A+	mild	500 units	2 days/week
15	24/M	36	A	VIII	B+	mild	500 units	2 days/week
16	15/M	46	A	VIII	AB+	mild	500 units	2 days/week
17	43/M	38	A	VIII	AB+	mild	500 units	3 days/week
18	29/M	46	A	VIII	B+	moderate	1000 units	3 days/week
19	28/M	36	A	VIII	AB+	Severe	1500 units	3 days/week
20	26/M	47	A	VIII	A+	Severe	1000 units	Once in every 2 days
21	33/M	46	A	VIII	A+	Severe	1000 units	Once in every 2 days
22	35/M	46	A	VIII	A+	mild	500 units	2 days/week
23	14/M	36	A	VIII	A+	mild	500 units	2 days/week
24	18/M	46	A	VIII	AB+	mild	500 units	2 days/week
25	27/M	36	A	VIII	AB+	mild	1000 units	2 days/week
26	14/M	36	A	VIII	B+	mild	500 units	2 days/week
27	20/M	47	A	VIII	B+	Severe	1000 units	3 days/week
28	22/M	46	A	VIII	B+	mild	500 units	2 days/week
29	18/M	36	A	VIII	AB+	moderate	1000 units	2 days/week
30	20/M	46	A	VIII	AB+	mild	1000 units	2 days/week

The degree of severity is varied: The normal range of factor VIII is 50–100 IU/dl (severe (<1 IU/dL per kg), moderate (1–5 IU/dL per Kg), mild (6–40 IU/dL per kg), and carriers who are treated as mild hemophiliacs if the factor level is <50 IU/dL per kg).

**Table 2:** Details of blood transfusion and iron-chelation therapy in thalassemic patients.

S No.	Age/Sex	Tooth No.	Type of Thalassemia	Blood Transfusion	Patient with Iron-Chelation Therapy	Patient without Iron-Chelation Therapy	Splenectomy	Disease Onset
1	48/M	47	B-TI	Once a year		✓		4 years old
2	50/F	46	B-TI	Once in 6 months	✓			6 years old
3	38/M	36	B-TMi	No		✓		13 years old
4	44/F	37	B-TMi	No		✓		16 years old
5	29/F	46	B-TM	Every 2 weeks	✓			2 years old
6	20/M	37	B-TM	Every 2 weeks	✓			At birth
7	35/F	46	B-TM	Every 2 weeks	✓		✓	2 monthsold
8	18/M	48	B-TM	Every 3 weeks	✓			At birth
9	16/M	47	B-TM	Every 3 weeks	✓			6 months old
10	30/M	47	B-TM	Every 2 weeks	✓		✓	5 months old
11	15/M	36	B-TM	Every 3 weeks	✓			6 months old
12	19/M	36	B-TM	Every 3 weeks	✓			2 months old
13	27/M	36	B-TM	Every 2 weeks	✓		✓	At birth
14	26/M	48	B-TM	Every 2 weeks	✓		✓	At birth
15	15/M	46	B-TM	Every 3 weeks	✓			2 months old
16	58/F	37	B-TMi	No		✓		18 years old
17	14/F	36	B-TM	Every 3 weeks	✓			
18	17/M	36	B-TM	Every 3 weeks	✓			4 months old
19	20/M	47	B-TM	Every 3 weeks	✓			5 months old
20	32/F	46	B-TI	Once in 6 months		✓		10 months
21	33/F	36	B-TI	Once in 6 months	✓			5 years old
22	38/M	37	B-TI	Once a year	✓			7 years old
23	41/F	46	B-TI	Once a year	✓			4 years old
24	33/F	38	B-TI	Once in 6 months	✓			4yrs old
25	30/M	46	B-TM	Every 2 weeks	✓		✓	At birth
26	26/F	36	B-TM	Every 2 weeks	✓			2 months old
27	32/F	36	B-TM	Every 2 weeks	✓		✓	5 months old
28	31/M	46	B-TM	Every 2 weeks	✓			8 months old
29	34/M	46	B-TMi	No		✓		10 years old
30	22/F	37	B-TM	Every 2 weeks	✓			2 months old

B-TMi: Beta-Thalassemia minor, B-TI: Beta-Thalassemic intermedia, B-TM: Beta-Thalassemia major.

**Table 3:** Age distribution by groups.

Age Group Years	Hemophilic		Thalassemic		Control	
	No.	%	No.	%	No.	%
14–20	14	46.7	9	30	6	20
21–29	11	36.7	5	16.7	7	23.3
30–39	4	13.3	11	36.7	10	33.3
≥40	1	3.3	5	16.7	7	23.3
Total	30	100	30	100	30	100
Mean ± SD (Years)	22.93 ± 7.17		29.7 ± 11.24		30.33 ± 10.4	

**Table 4:** Gender distribution by groups.

Group	Male		Female		Total	
	No.	%	No.	%	No.	%
Hemophilic	28	93.3	2	6.7	30	100
Thalassemic	17	56.7	13	43.3	30	100
Control	13	43.3	17	56.7	30	100
Total	58	64.4	32	35.6	90	100

**Table 5:** Comparison of Onset time (in sec) of LA.

	Groups	N	Mean	Std. Deviation	Std. Error	ANOVA (*p* Value)
**Onset time of LA**	Hemophilia	30	45.967	34.3215	6.2662	0.56
	Thalassemia	30	42.400	23.3631	4.2655	
	Control	30	38.967	12.8532	2.3467	
	Total	90	42.444	24.9755	2.6327	

**Table 6:** Volume of anesthesia increases within the groups.

Group	Yes		No		Total	
	No.	%	No.	%	No.	%
Hemophilic	6	20	24	80	30	100
Thalassemic	4	13.3	26	86.7	30	100
Control	4	13.3	26	86.7	30	100
Total	14	15.6	76	84.4	90	100

**Table 7:** Comparison of the VAS at different intervals among the groups.

		N	Mean	Std. Deviation	Std. Error	ANOVA (*p* Value)
**Pre-op VAS**	Hemophilia	30	7.467	1.6554	0.3022	0.17
	Thalassemia	30	8.000	1.3646	0.2491	
	Control	30	8.133	1.2521	0.2286	
	Total	90	7.867	1.4472	0.1525	
**LA-VAS**	Hemophilia	30	2.733	2.5722	0.4696	0.16
	Thalassemia	30	3.300	2.1838	0.3987	
	Control	30	2.133	2.2854	0.4173	
	Total	90	2.722	2.3751	0.2504	
**PE-VAS**	Hemophilia	30	1.267	2.0833	0.3804	0.82
	Thalassemia	30	1.000	1.8004	0.3287	
	Control	30	1.000	1.7019	0.3107	
	Total	90	1.089	1.8521	0.1952	
**CI-VAS**	Hemophilia	30	0.100	0.3051	0.0557	0.55
	Thalassemia	30	0.100	0.3051	0.0557	
	Control	30	0.033	0.1826	0.0333	
	Total	90	0.078	0.2693	0.0284	

**Table 8:** Comparison of pain perception at various intervals.

		N	Mean	Std. Deviation	Std. Error	ANOVA (*p* Value)
**Pre-op VAS**	Mild	30	1.000	0.0000	0.0000	
	Moderate	30	1.000	0.0000	0.0000	
	Severe	30	1.000	0.0000	0.0000	
	Total	90	1.000	0.0000	0.0000	
**LA-VAS**	Mild	30	0.600	0.4983	0.0910	0.048 ^†^
	Moderate	30	0.700	0.4661	0.0851	
	Severe	30	0.833	0.3790	0.0692	
	Total	90	0.711	0.4558	0.0480	
**PE-VAS**	Mild	30	0.567	0.7279	0.1329	0.926
	Moderate	30	0.567	0.8172	0.1492	
	Severe	30	0.500	0.7311	0.1335	
	Total	90	0.544	0.7519	0.0793	
**CI-VAS**	Mild	30	0.033	0.1826	0.0333	0.547
	Moderate	30	0.100	0.3051	0.0557	
	Severe	30	0.100	0.3051	0.0557	
	Total	90	0.078	0.2693	0.0284	

† Significant at *p* < 0.05.

**Table 9:** Correlation between the VAS and onset time.

Model	Coefficients				
	Unstandardized Coefficients		Standardized Coefficients	t	Sig.
	B	Std. Error	Beta		
Pre-op VAS	7.300	0.296	0.230	24.696	0.000
LA-VAS	1.368	0.470	0.335	3.341	0.001
PE-VAS	−0.253	0.352	0.426	−0.720	0.473

## Data Availability

Requested data from corresponding author.
